# Fabrication of Size-Tunable Metallic Nanoparticles Using Plasmid DNA as a Biomolecular Reactor

**DOI:** 10.3390/nano1010064

**Published:** 2011-10-21

**Authors:** Jacopo Samson, Irene Piscopo, Alex Yampolski, Patrick Nahirney, Andrea Parpas, Amit Aggarwal, Raihan Saleh, Charles Michael Drain

**Affiliations:** 1 Department of Chemistry, Hunter College of the City University of New York, 695 Park Avenue, New York, NY 10065, USA; E-Mails: jsamson@hunter.cuny.edu (J.S.); yampolsa@gmail.com (A.Y.); aparpas@hunter.cuny.edu (A.P.); amitashin@yahoo.co.in (A.A.); raihan.saleh@gmail.com (R.S.); 2 EM Consulting, 57 Soundview Drive, Huntington, NY 11743, USA; E-Mail: irene.piscopo@gmail.com; 3 Division of Medical Sciences, University of Victoria, Victoria, BC V8W 2Y2, Canada; E-Mail: nahirney@uvic.ca; 4 The Rockefeller University, 695 Park Avenue, New York, NY 10065, USA

**Keywords:** plasmid DNA, biomolecular reactor, gold, silver, palladium, chromium nanoparticles, green synthesis

## Abstract

Plasmid DNA can be used as a template to yield gold, palladium, silver, and chromium nanoparticles of different sizes based on variations in incubation time at 70 °C with gold phosphine complexes, with the acetates of silver or palladium, or chromium acetylacetonate. The employment of mild synthetic conditions, minimal procedural steps, and aqueous solvents makes this method environmentally greener and ensures general feasibility. The use of plasmids exploits the capabilities of the biotechnology industry as a source of nanoreactor materials.

## 1. Introduction

Gold nanoparticles (Au NPs) and other metallic based nanoparticles (e.g., nickel, silver, palladium, chromium) are synthesized by a variety of methods because of their wide range of potential applications, e.g., drug delivery systems, catalysts, optical sensors, and antimicrobial agents [1–8]. However, the harsh conditions employed in several synthetic approaches has motivated researchers to investigate milder routes to obtain metal NP [9]. Organic molecules and inorganic molds can be used to control NP growth and dimensions [8]. Though confusing, the literature refers to “templates” both as sites where NP are formed and as a means to organize the NP into hierarchal patterns. The formation of inorganic NP in nature, such as the complex morphologies of carbonate [10], ferritin [11–13], and other NP [14] has inspired significant efforts to form and organize NP using biological systems[15]. Macromolecules can serve as a scaffold where NP form and bind, or as a nanoreactor (mold) that determines the size of the forming NP. In both cases nucleation can be on the macromolecule. Biological systems such as proteins [16], viruses [17], and plasmid DNA [18] were shown to be successful scaffolds or molds enabling milder pathways for the formation of NP. To date, the methodologies employing biological reagents present other drawbacks such as poor size tunability, broad dispersity, and limited shape control. The tendency of cationic gold to disproportionate in aqueous solutions [19], and issues centered on stabilizing metallic NPs further complicate synthetic methods based on biological macromolecules.

We previously demonstrated that the toroidal topology of plasmid DNA is a viable mold for the formation of gold (Au), nickel (Ni) and cobalt (Co) NP. Plasmid DNA is readily available, inexpensive, and the size of the NP is tunable based on length of the biopolymer and the inner diameter of the toroid [18]. The size of the NP formed also is dictated by several varying parameters that influence the particle formation mechanism, e.g., G-C *versus* A-T content and different degrees of topological purity of the plasmid suspensions. The previous report used UV light to catalyze photo-oxidative degradation of the plasmid DNA with the concomitant reduction of the metal ions. Thus, the reproducibility is also dependent on maintaining a specific energy flux during the irradiation time [18].

**Figure 1.**
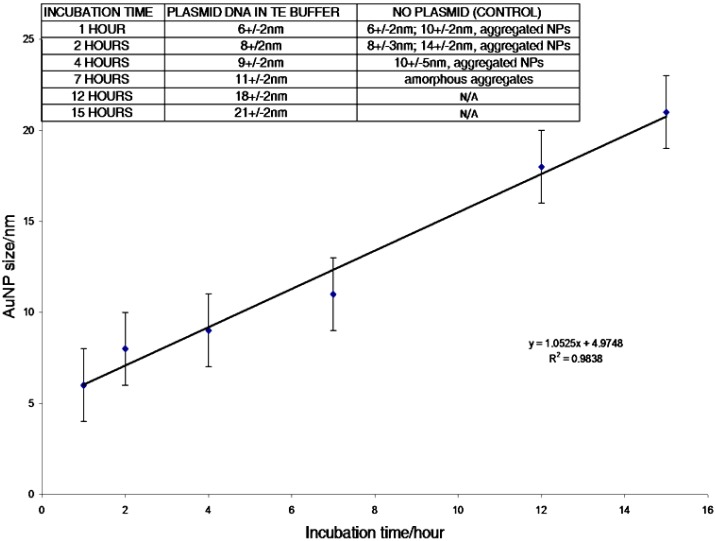
Incubation time of the gold phosphine precursor with plasmid DNA versus NP size determined by TEM analysis.

Herein, we present a complementary synthetic method based on a kinetic approach wherein the plasmid DNA (pcDNA 3.1(+)/GFP, approx. 6 kbps) acts as a nanoreactor to initiate and control the formation of Au and other metallic NPs by incubation at elevated temperatures. This is an easy to follow procedure that requires less energy, time, and reagents than many other templating systems [14]. The size of the Au NPs can be controlled by varying the incubation times (Figure 1). Similar procedures allow the preparation of other NP including silver (Ag), palladium (Pd), and chromium (Cr). In this method the metal ions are reduced by oxidative degradation of the amine buffer and/or the DNA at elevated temperatures, and disproportionation reactions for Au, thereby obviating the need for auxiliary reducing agents such as hydrazine and sodium borohydride.

## 2. Experimental Section

### 2.1. Instrumentation and Materials

#### 2.1.1. UV-Visible

A Beckman Coulter DU800 spectrophotometer was used where 50 μL of each sample was loaded into the 8 mm path length cell. PCR Eppendorf tubes were used. Silver acetate, cadmium acetonalacetonate, palladium acetate, and tris(hydroxymethyl)-aminomethane (Tris) buffers were from Sigma-Aldrich. TE (Tris/EDTA) buffer was from Quiagen and made of Tris (10 mM) and ethylenediaminetetraacetic acid (EDTA, 1 mM) at pH 8 (structures of Tris and EDTA in SM-Scheme 1). A Lab-Line Multi block heater was used and samples were incubated in the dark under an aluminum foil sheet.

#### 2.1.2. Transmission Electron Microscopy (TEM)

All data were collected at 120 kV on a Tecnai TEM (FEI) at the eucentric height to ensure that all measurements and electron diffraction (ED) data were accurate for both collection and comparison. The electron diffraction patterns were collected in the microprobe or nanoprobe mode depending on the size of the area to be analyzed. A 7 μL drop of the aqueous DNA suspension was placed on a 200 mesh carbon coated copper grid, (TED Pella Inc., Redding, California, USA), and allowed to dry for 5 minutes in the dark. The remaining liquid was “whisked” away using a filter paper. The control samples were prepared in the same way in the absence of DNA. The spherical shape of particles was determined by eucentric tilting (over at least an 80° range) over the particles. Average particle sizes were determined by counting 50 particles from the TEM images for the NP samples using “imageJ” software (NIH).

#### 2.1.3. Gel Electrophoresis

0.8% agarose gels were freshly prepared by dissolving 0.4 g of agarose, purchased (Sigma) in TE buffer (1×). 2.5 μL of ethidium bromide was added when the mixture was still in the liquid phase. After solidification, 10 μL of specific samples were loaded in each of the 10 wells and the gel was run at 85–90 V for 75 min.

#### 2.1.4. Plasmid DNA

pcDNA 3.1(+)/GFP was amplified following Quiagen protocols. In order to determine plasmid size, a sample of each circular plasmid DNA was incubated with ECORI restriction enzyme and linearized. After running on a 0.8% agarose gel, the following sizes were deduced from 1 kb standard ladder (Biolabs): pcDNA 3.1(+)/GFP: ∼6 kbp (3960 kDa); pMSCV: ∼7 kbp; pBABEc: ∼6 kbp.

#### 2.1.5. Metal Nanoparticles

**Figure 2.**
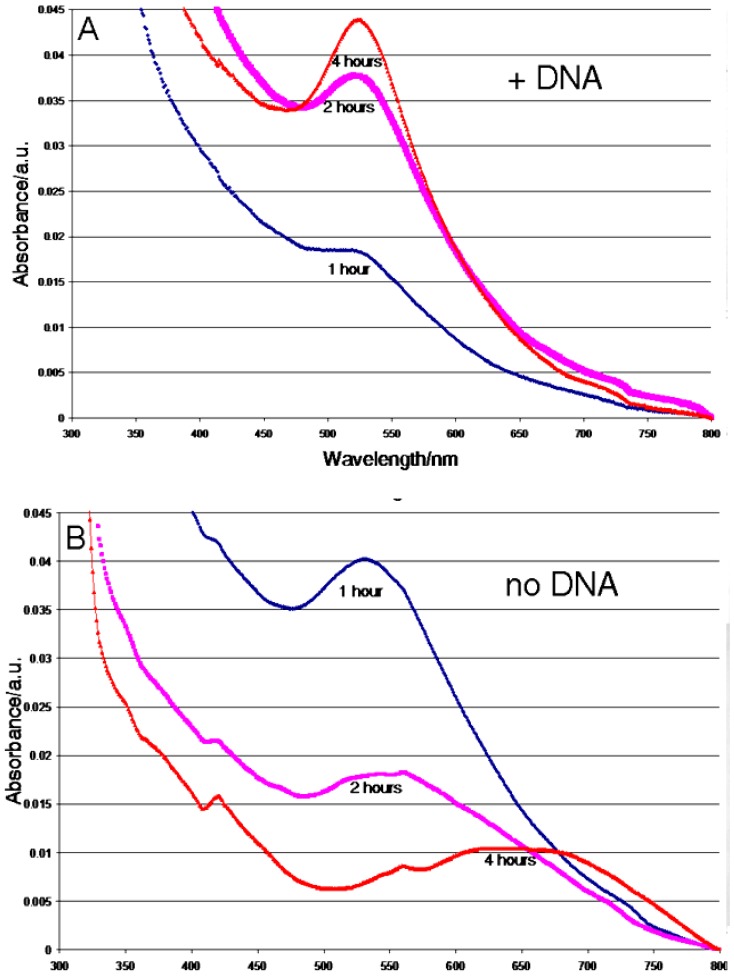

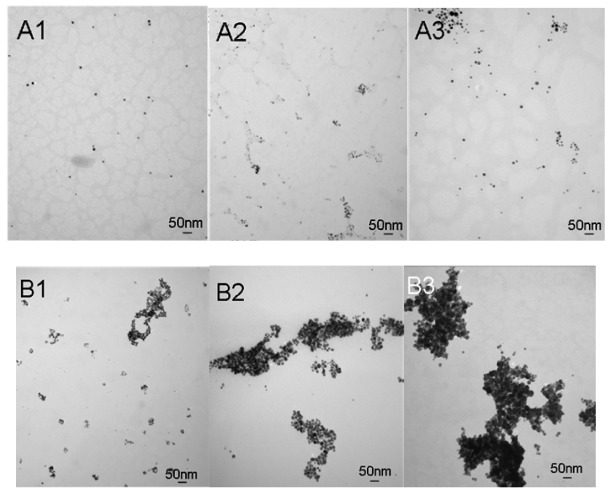
(**A**) The UV-visible spectra of the Au nanoparticles formed by heating the solution containing the DNA in TE buffer and the gold phosphine at 70 °C in the dark for 1 h (blue line), 2 h (pink line), and 4 h (red line). Transmission electron microscopy images were taken of these three samples. (**A1**) TEM image of the sample heated for 1 h, (**A2**) TEM image of the sample heated for 2 h, and (**A3**) TEM image of the sample heated for 4 h. (**B**) The UV-visible spectra of the Au nanoparticles formed in control reactions by heating the solution containing only the gold phosphine and the TE buffer at 70 °C in the dark for 1 h (blue line), 2 h (pink line), and 4 h (red line). Transmission electron microscopy images were taken of these three control samples. (**B1**) TEM image of the control sample heated for 1 h, (**B2**) TEM image of the control sample heated for 2 h, and (**B3**) TEM image of the control sample heated for 4 h.

**Figure 3.**
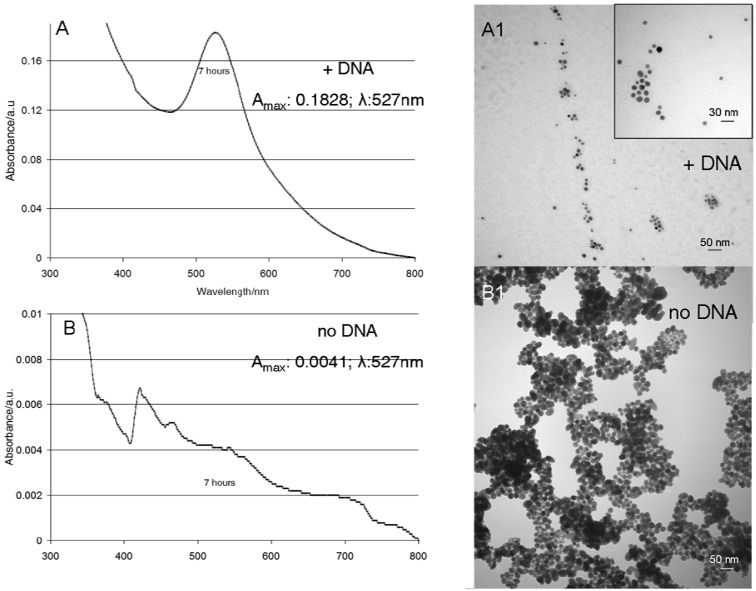
(**A**) UV-visible spectrum and corresponding TEM image (A1) of DNA samples incubated with gold phosphine solution for 7 h at 70 °C in the dark; Inset panel: higher magnification of sample in A1. (**B**) UV-visible spectrum and corresponding TEM image (B1) of TE buffer control incubated with gold phosphine solution under the same conditions.

In a 200 μL PCR Eppendorf tube, 60 μL of a DNA suspension (35 ng/μL, ∼5 × 10^−13^ moles, in TE buffer, pH 8) was incubated with 10 μL of a solution containing 0.5 wt % chloro trimethylphosphine-gold (I) in acetone (1.6 × 10^−4^ M, 1.6 × 10^−9^ moles, referred as the gold phosphine solution) in the dark at 70 °C in a programmable block heater. This method yielded the formation of 6, 8, 9, 11, 18, and 21 nm diameter Au NPs after 1, 2, 4, 7, 12, and 15 h of DNA/metal cations incubation, respectively (Figures 1–3). When the incubation time was extended to 21 hours, the water of the DNA suspension evaporated and condensed on the lid of the PCR Eppendorf tube, causing the DNA, as well as other randomly shaped gold structures present in solution, to aggregate at the bottom of the tube. This aggregation of amorphous DNA and Au material was confirmed by UV-visible spectroscopy [20] (supplementary material). Control experiments with TE buffer and gold phosphine solution were run in the absence of DNA for 1, 2, 4, and 7 h at 70 °C, respectively (Figures 2 and 3) and result in randomly sized aggregates. A product yield of 69% was found for the Au NP formed in the presence of the plasmid, and was calculated via equation 1 below (also see Figure SM-1 for detailed calculations) [21]. For formation of the other metallic NP, ethanol solutions containing 0.5 wt % of palladium acetate, chromium acetylacetonate, or silver acetate were used.

## 3. Results and Discussion

The synthetic parameters and trends in NP formation were evaluated with one plasmid DNA and gold phosphine, and these data were used as a guide to fabricate NP of Ag, Pd, and Cr. UV-visible spectra of the solution from control experiments with only the gold phosphine and the TE buffer incubated for 1 h at 70 °C showed the presence of Au NP (Figure SM-2), and was similar to those with the plasmid DNA under the same conditions, suggesting that the TE buffer may act as an initial seeding entity. Other amines are known to serve as reducing agents [22–25], and the DNA can serve as a source of electrons at elevated temperatures. Preparations with non-amine buffers such as phosphate do not yield Au NP. UV-visible spectra from the 2 h and the 4 h control experiments showed a progressive diminishment of the peak corresponding to the Au NP absorption as well as an increasing red shift, indicating the formation of larger and more amorphous Au materials. These latter controls confirm that the plasmid DNA is responsible for both the size control and the narrow distribution of the fabricated Au NPs (Figures 2 and 3). Furthermore, in the absence of DNA, amorphous gold aggregates were observed after 7 h of incubation (Figure 3). The role of the DNA as a mold also was deduced by comparing the UV-visible spectra with an analysis of the fate of the DNA by gel electrophoresis (Figures 2, 3 and SM-3, SM-4, SM-5). After a four hour incubation experiment, the DNA gels showed a substantial modification of the initial plasmid topology and eventually the DNA is not observed (Figure SM-2, compare lanes 2 and 10). The UV-visible spectra indicate increased formation of the Au NP with time, but not an increase in NP size (Figure SM-1). The ratio of initial DNA condensation states, supercoiled *versus* relaxed, dictates the dispersity of the NP products (Figure SM-3, lanes 2 and 5). Fabrication of narrowly dispersed particles was obtained concomitant with degradation of the DNA, as observed starting around two hours of incubation time with the gold phosphine (Figure SM-3, lanes 2 and 5). Our hypothesis is that degraded DNA segments maintain particle dispersion by inhibiting aggregation during incubation, although we cannot exclude the possibility that the DNA from the plasmid surrounds the particle (Figures SM-6 and SM-7). The maximum amount of Au NP is reached by seven hours of incubation as revealed by comparison of the UV-visible absorption peaks shown in Figure 3(A) and 3(B) and the corresponding TEM images. The size tunability of the Au NP *versus* incubation time was verified by TEM measurements, and is supported by the corresponding UV-visible spectra (Figures 2 and 3).

**Figure 4.**
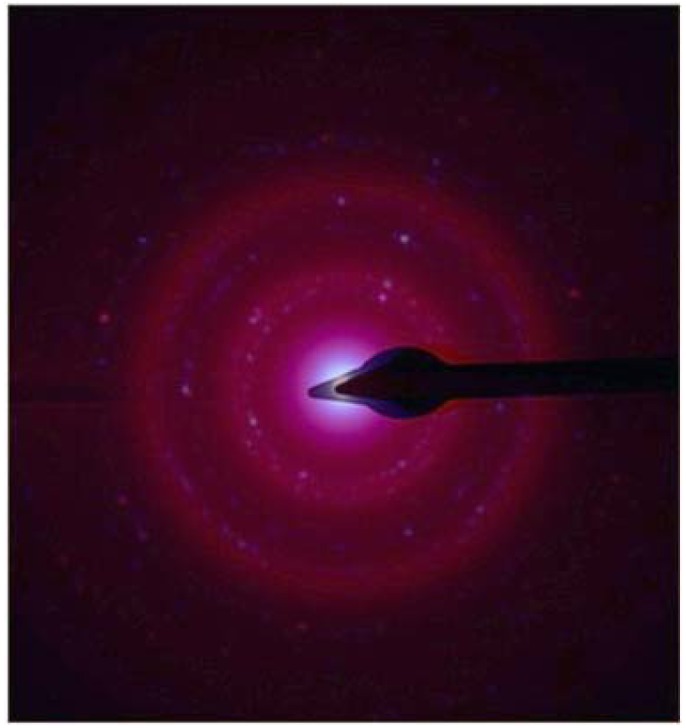
ED pattern of metallic gold standard solution (blue) superimposed on the experimental ED pattern obtained from Au NPs analysis (red) where the overlap indicates the DNA template prepared sample is metallic Au.

The metallic nature of the Au NPs was confirmed by superimposing the ED pattern obtained from the experimental samples with that of a metallic gold standard (Figure 4) [26]. The spherical shape of the Au NP were determined by eucentric tilting (see experimental and supplementary material). The relative proportions (∼3200:1 Au:plasmid or 1:2 Au:base pairs) and concentrations of plasmid DNA and gold salt outlined in the experimental section reproducibly yield the narrow dispersity NP. These conditions were found empirically. With the same amount of gold salt, dilution of the plasmid DNA concentration by 50-fold results in a broadening of the UV-visible spectra of the Au NP indicating a much broader distribution of NP. A 5-fold increase of plasmid DNA concentration yielded much fewer particles (Figure SM-8), perhaps because too much of the gold is bound by the plasmid thereby inhibiting NP growth (1:10 Au:base pair). Furthermore, the UV-visible spectra of Au NP from the 50 fold diluted sample containing plasmid DNA, was nearly identical to the spectra of the TE buffer/Au controls. The reaction does not yield NP when the solvent evaporates (Figure SM-9). These observations suggest that a balance between the relative quantities of the metal salts, the plasmid DNA, and the buffer are necessary to ensure the narrow dispersity of the NP. After about two hours, the size of the NP increases but the dispersity of the NP narrows, and this is reflected in the UV-visible spectra as a progressive red shift without broadening. This observation is consistent with previous studies correlating UV-visible spectra with NP size [20]. Nucleation of the nanoparticles initiating inside the toroid in the minor and major grooves of DNA, where cationic binding is often observed [27], may explain the size control of the Au NPs (see supplementary materials).

**Figure 5.**
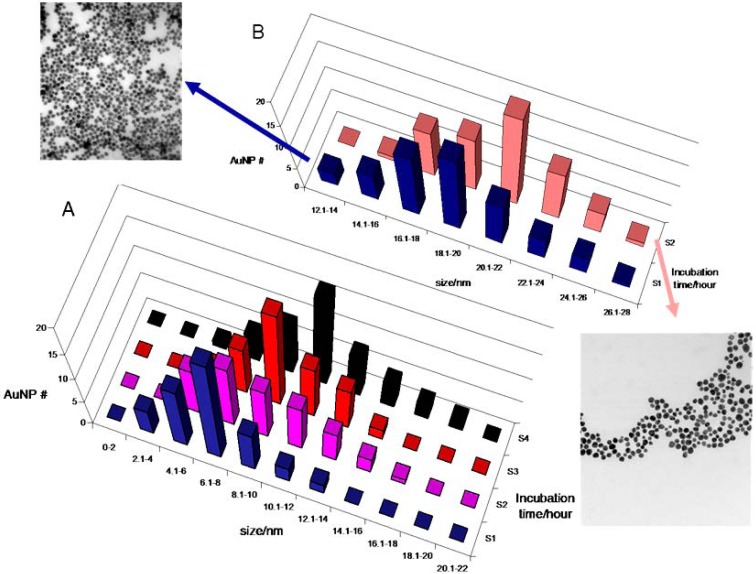
(**A**) Histogram of Au NP size distributions corresponding to samples incubated with plasmid DNA in TE buffer for 1 h (S1, blue), 2 h (S2, purple), 4 h (S3, red), and 7 h (S4, black). After 4 h there is a progressive narrowing of the distribution and an increase in particle size that is in agreement with the red shifts observed in the UV-visible spectra (Figures 1 and 2). (**B**) Histogram of Au NP sizes corresponding to samples incubated with plasmid DNA for 12 h (S1, dark blue) and 15 h (S2, pink). TEM images of the Au NP from 12 h (dark blue) and 15 h (pink) incubations at 70 °C.

**Figure 6.**
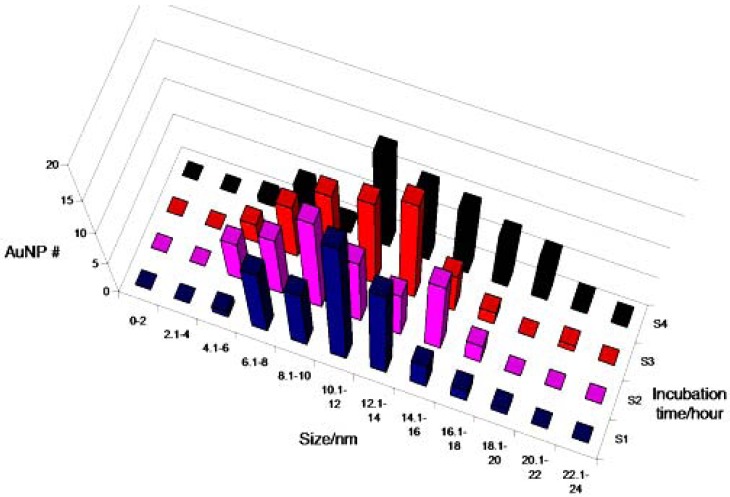
Histogram of NP size distributions of control samples in TE buffer without plasmid DNA incubated for 1 h (S1, blue), 2 h (S2, purple), 4 h (S3, red), and 7 h (S4, black). Despite the high level of aggregation, the particles were still counted individually. The level of particle aggregation was greatest in the 7 h sample (Figure 3). No correlation between the NP size and the incubation time was observed.

**Figure 7.**
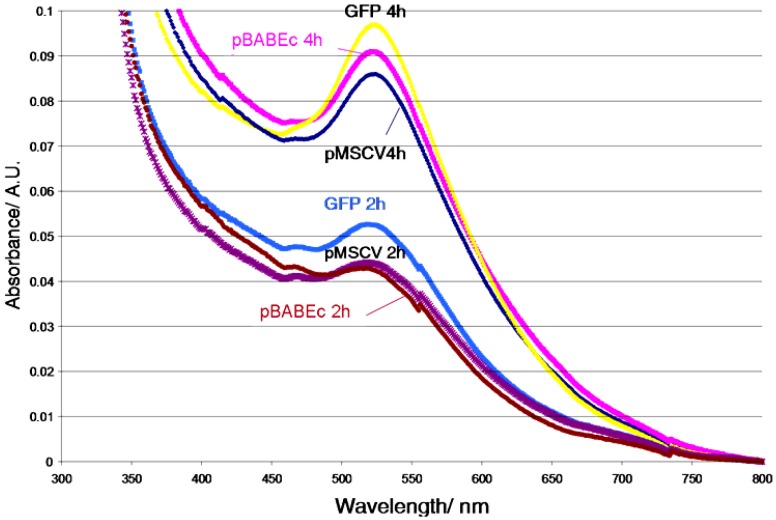
UV-visible spectra of three different plasmid DNA samples incubated in the dark at 70 °C for 2 and 4 h. The λ_max_ and half-width of the peaks indicate that these plasmids yield Au NPs with similar size and distribution. The spikes at ∼550 nm and ∼740 nm are instrumental artifacts.

The overall yield of the NP can be estimated from equation 1. After a typical 4 h incubation time at 70 °C, the yield is about 8%, but the yield increases to about 70% after two weeks at room temperature (Figure SM-1). This indicates that incubation at elevated temperatures initiates the NP formation process. Histograms of the size distribution of the Au NP under several conditions, and the control TE buffer are shown in Figures 5 and 6. UV-Visible spectra from Figure 7 show that different plasmid DNA constructs with similar base pair count (approximately 6kbps) yield narrowly dispersed gold nanoparticles indicating that a specific plasmid DNA is not necessary.

(1) $$\begin{array}{c} \text{Yield NP}:{\text{C}}_{\text{initial NP}}/{\text{C}}_{\text{synthesized NP}}\%\\ {\text{C}}_{\text{initial NP}}={\text{N}}_{\text{tot}}/{\text{N}}_{\text{A}}\;\text{V N}\\ {\text{C}}_{\text{synthesized NP}}=\text{A}\;/\;ɛ \end{array}$$

## 4. Ag, Pd, and Cr Nanoparticles

**Figure 8.**
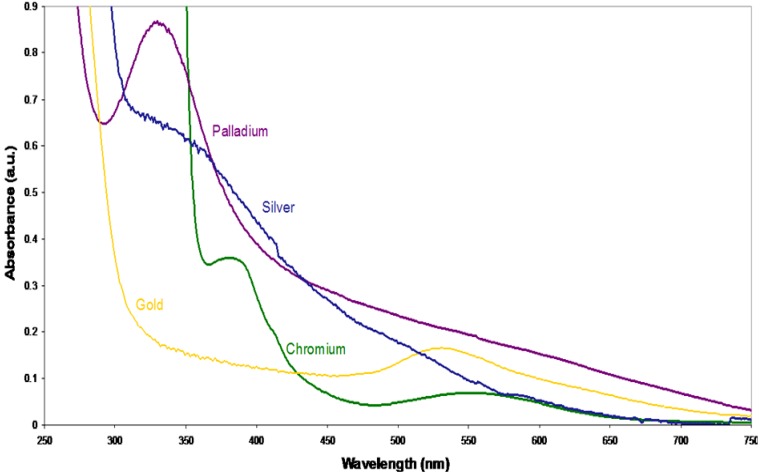
The UV-visible spectra of nanoparticles of palladium (purple), silver (blue), and chromium (green) compared to that of gold (yellow) are consistent with data reported previously.

**Figure 9.**
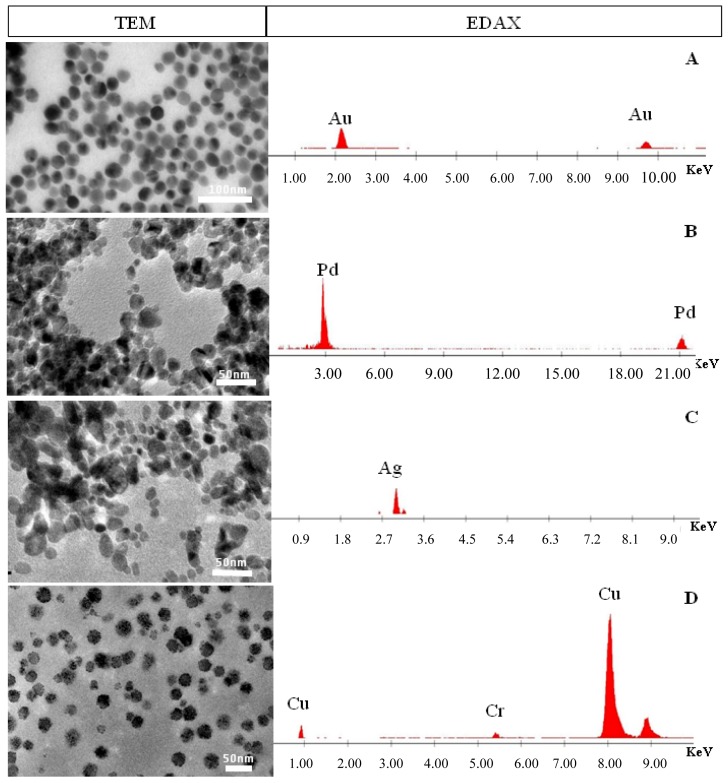
**Left column:** Representative TEM images of nanoparticles composed of different metals that were formed by heating a solution of the metal ion precursor with the plasmid DNA in Tris buffer at 70 °C for various times. The Au reaction was heated for 12 h, the Pd reaction was heated for 17 h, the Ag reaction was heated for 10 h, and the Cr reaction was heated for 10 h. From the top are the TEM of the Au, Pd, Ag, and Cr nanoparticles. **Right column:** EDAX Netcounts (sample area minus control area) spectra indicate the composition of the metallic nanoparticles found in the TEM images. From the top are the EDAX spectra of (**A**) Au, (**B**) Pd, (**C**) Ag. For the Cr nanoparticles (**D**), the Netcounts method was not used so the x-ray scattered lines from the carbon coated copper grid, show up in the spectrum (indicated by the Cu peak).

Metallic nanoparticles of Pd are widely used as catalysts for organic transformations and coupling reactions [28–30]. Silver nanoparticles are exploited as sensors in surface enhanced Raman and other methods [31–35]. Chromium nanoparticles are proposed for a variety of photonics applications, including in solar energy harvesting [36]. Therefore, facile and greener synthetic methods resulting in narrowly dispersed nanoparticles of these metals are of significant interest. The same procedures and considerations are used to make metallic nanoparticles of Pd, Ag, and Cr. Using our optimized conditions from the above Au NP experiments, the plasmid DNA is incubated at 70 °C between 10 h and 17 h depending on the metal ion used. These systems are characterized by UV-visible spectroscopy, TEM, and energy dispersive x-ray microanalysis (EDAX) (Figures 8 and 9). These data are consistent with those reported in the aforementioned literature.

The mechanism of metallic NP formation inside a variety of organic and biological structures has been discussed in terms of increased concentration of nucleation sites on the interior surface *versus* the exterior surface, the topology of the nanoreactor, and in terms of the reaction conditions [37,38].

## 5. Conclusions

The results demonstrate the ease of synthesis of size tunable nearly spherical nanoparticles of Au, and narrowly dispersed NP of several types of metals, that exploits the toroid topology of plasmid DNA as a mold in concert with the slow, temperature controllable oxidation of an amine buffer. While our previous report used UV light to catalyze the oxidation of the DNA and concomitant reduction of the metal cations [18], herein we employ the application of heat to initiate oxidation of the TE buffer to reduce the metal cations. The multi block heater ensures exact temperature control over a predetermined amount of time while using minimal energy. The stabilization of the metallic NP is mediated by the plasmid DNA fragments. These mild synthetic conditions makes this method environmentally more sustainable, while the minimal steps and the variety of possible plasmids with differences in topology and size enables widespread applications and feasibility for the synthesis of metallic NPs.

## Supplementary Material
