# Supplementary material for: Fabrication of Size-Tunable Metallic Nanoparticles Using Plasmid DNA as a Biomolecular Reactor
Source: Nanomaterials (Basel). 2011 Oct 21;1(1):64–78. doi: 10.3390/nano1010064 (PMC5315049; doi:10.3390/nano1010064)

Nanomaterials **2011**, *1*, 64-78; doi:10.3390/nano1010064

OPEN ACCESS

Nanomaterials

ISSN 2079-4991

www.mdpi.com/journal/nanomaterials

Article

Fabrication of Size-Tunable Metallic Nanoparticles Using Plasmid DNA as a Biomolecular Reactor

Jacopo Samson [1], Irene Piscopo [2], Alex Yampolski [1], Patrick Nahirney [3], Andrea Parpas [1], Amit Aggarwal [1], Raihan Saleh [1] and Charles Michael Drain [1,4,*]

[1] Department of Chemistry, Hunter College of the City University of New York, 695 Park Avenue, New York, NY 10065, USA; E-Mails: jsamson@hunter.cuny.edu (J.S.); yampolsa@gmail.com (A.Y.); aparpas@hunter.cuny.edu (A.P.); amitashin@yahoo.co.in (A.A.); raihan.saleh@gmail.com (R.S.)

[2] EM Consulting, 57 Soundview Drive, Huntington, NY 11743, USA; E-Mail: irene.piscopo@gmail.com

[3] Division of Medical Sciences, University of Victoria, Victoria, BC V8W 2Y2, Canada; E-Mail: nahirney@uvic.ca

[4] The Rockefeller University, 695 Park Avenue, New York, NY 10065, USA

* Author to whom correspondence should be addressed; E-Mail: cdrain@hunter.cuny.edu; Tel.: +1-212-650-3791; Fax: +1-212-722-5332.

Supplementary Material

Scheme SM-1. structure of EDTA and Tris P2
Figure SM-1. UV-is of a typical 4 h incubation time sample for yield calculations P2
Figure SM-2. UV-vis spectra of Au NP and from control reactions P3
Figure SM-3. Gel electrophoresis of DNA incubated with gold phosphine P3
Figure SM-4. UV-vis spectra of GFP and GFPr plasmids P4
Figure SM-5. UV-vis spectra of longer incubation times of samples from SM-4 P5
Figure SM-6. UV-vis of samples precipitated with isopropyl alcohol P5
Figure SM-7. TEM and histogram of NP after 30 min incubation for mechanism P6
Figure SM-8. UV-vis of 50 fold diluted GFP incubated with gold phosphine for 4 h P7
Figure SM-9. UV-vis of 21 h-DNA suspension and gold phosphine solution P7

SM-Scheme 1. Structure of TRIS, left, and EDTA, right.

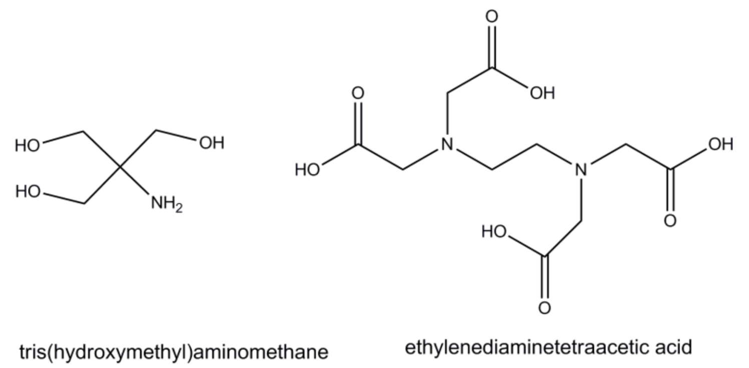

Figure SM-1. (**A**) UV-visible spectra of samples after incubation for 4 h. The samples were allowed to sit at room temperature in the dark for a given amount of time. The yield of the reaction increases up to 69% after 2 weeks. The absence of a red shift confirms that the particles in solution neither re-aggregate nor collapse through time. A high stability in solution is therefore maintained over the course of weeks. (**B**) shows the yields calculated based on the absorbance values corresponding to the UV-visible spectra.

A

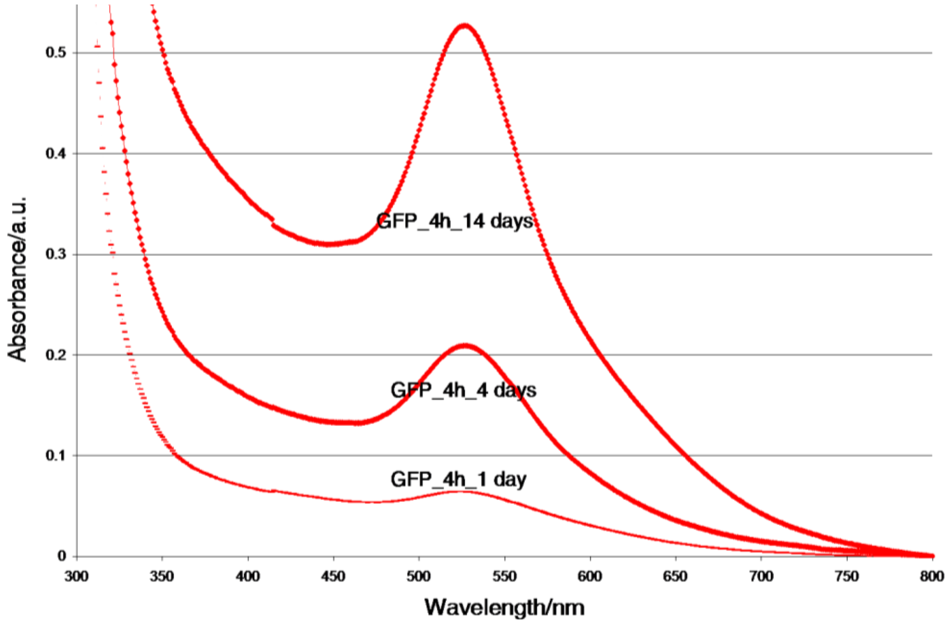

B

AuNP size	Abs/A.u.	[NP*]	Yield NP/%
9nm	0.0644	4.32×10^{-6} M	8%
	0.2092	1.40×10^{-5}	27%
	0.5267	3.53×10^{-5} M	69%

* $C_{\text{initial NP}} = N_{tot}/N_A$ V N = 5.14×10^{-5}

N_{tot} calculated from initial 0.016g of Au = $8.11 \times 10^{-5} \times N_A = 4.88 \times 10^{19}$

$N_A = 6.02 \times 10^{23}$

$N = 30.89602\ D^3 = 22523$

Since the $C_{\text{initial NP}}$ is 3444 times bigger than the reference on Table, the ϵ has to be 3444 smaller than the reference (= 1.49×10^4)

$C_{\text{synthesized NP}} = 0.2092/\ 1 \times 1.49 \times 10^4 = 1.40 \times 10^{-5}$

Yield NP: $C_{\text{inintial NP}} / C_{\text{synthesized NP}}$ %

Figure SM-2. UV-visible spectra of control experiments incubated for 1 h and 2 h at 70 °C in the dark (water: green; TRIS: light blue; PBS: purple; EDTA: orange). The UV-visible spectra of the Tris controls (corresponding to the incubation times of 1 h and 2 h) resemble the spectra of the TE buffer controls corresponding to the same incubation times (Figure 2B) as well as the 1 h incubation DNA/gold (Figure 2A).

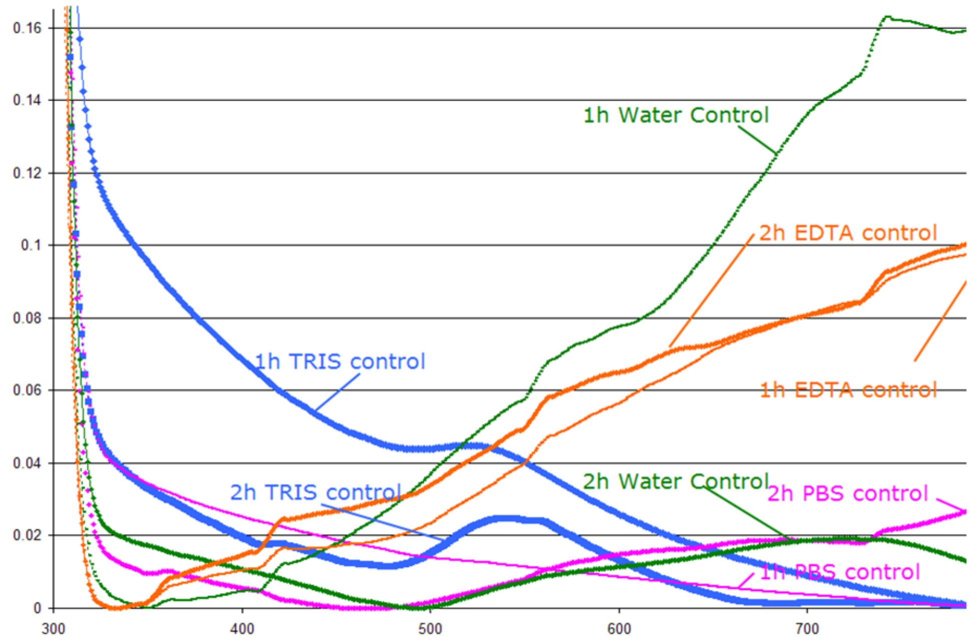

Figure SM-3. 0.8% agarose gel electrophoresis (GE) of plasmid DNA incubated with gold phosphine solution at different times. The gels were taken on different days (Panel A: same day; Panel B: the next day).

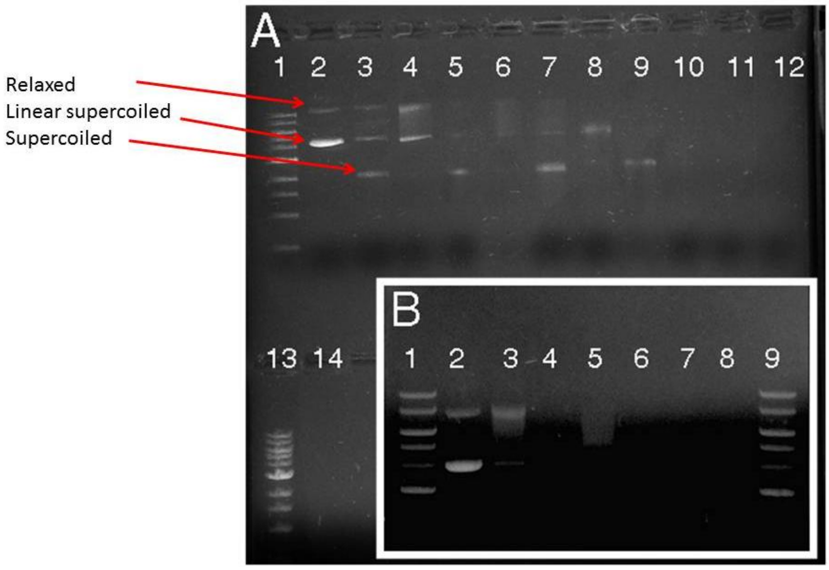

Panel A→ Lane 1: 1 kb ladder; Lane 2: naked pCDNA 3.1 (+)/GFP (GFP); Lane 3: naked pCDNA 3.1 (+)/GFP with rearranged initial topologies (GFPr); Lane 4: GFP after 30 min incubation time; Lane 5: GFPr after 30 min incubation time; Lane 6: GFP after 1 hour incubation time; Lane 7: GFPr after 1 hour incubation time; Lane 8: GFP after 2 hour incubation time. Lane 9: GFPr after 2 hour incubation

time; Lane 10: GFP after 4 hour incubation time; Lane 11: GFPr after 4 hour incubation time; Lane 12: GFP after 7 hour incubation time; Lane 13: 1 kb ladder; Lane 14: GFPr after 7 hour incubation time.

Panel B→ 1: 1 kb ladder; Lane 2: Naked pCDNA 3.1 (+)/GFP (GFP); Lane 3: GFP after 30 min incubation time; Lane 4: GFPr after 30 min incubation time; Lane 5: GFP after 1 hour incubation time; Lane 6: GFPr after 1 hour incubation time; Lane 7: GFP after 2 hour incubation time. Lane 8: GFPr after 2 hour incubation time; Lane 9: 1 kb ladder.

Starting from the fourth hour of incubation, either total degradation into DNA segments, or wrapping of DNA template around NP occurs thereby quenching the ethidium bromide, (undetectable by GE: lane 10, 11, 12, and 14). This shows a narrower particle distribution when GFP is the starting templating agent (see UV-visible spectra from Figures 2 and 3). The presence of the small DNA segments act as an anti-aggregating agent (Figure SM-6). Furthermore from lanes 8 and 9 (Panel A) we observed the presence of two different main DNA topologies (after 2 hour incubation time) in solution which yielded nanoparticles of similar size and distribution (compare UV-Vis spectra of GFP 2 h Vs GFPr 2 h from Figure SM-4). This confirms that DNA acts as a template and the presence of a specific topology (e.g. toroidal or highly condensed), is not necessary to drive the particle formation. On the other hand there are two great advantages in having a well defined initial DNA topological ratio: 1) the particles are more stable in solution and do not tend to re-aggregate (GFP 2 h + overnight vs. GFPr 2 h + overnight, Figure SM-4); 2) A trend of more narrowly distributed NP formation is ensured throughout longer incubation times of 4 and 7 (Figure SM-5).

Figure SM-4. Samples from Figure SM-3 lane 8 (GFP 2 h) and lane 9 (GFPr 2 h) incubated with gold phosphine at 70 ^{o}C for 2 hours. Spectra "GFP 2 h + overnight" and "GFPr 2 h + overnight" were taken the day after to investigate the effect of initial topologies on the particle formation. Changes in initial topologies (Figure SM-3, Panel A, lanes 2 & 3) resulted in no significant variation in particle size observed by no red shift. The size distribution is narrower when there is less of the supercoiled GFPr (brown and green lines), was utilized as initial template. This trend continues to be exhibited through longer incubation times (Figure SM-5).

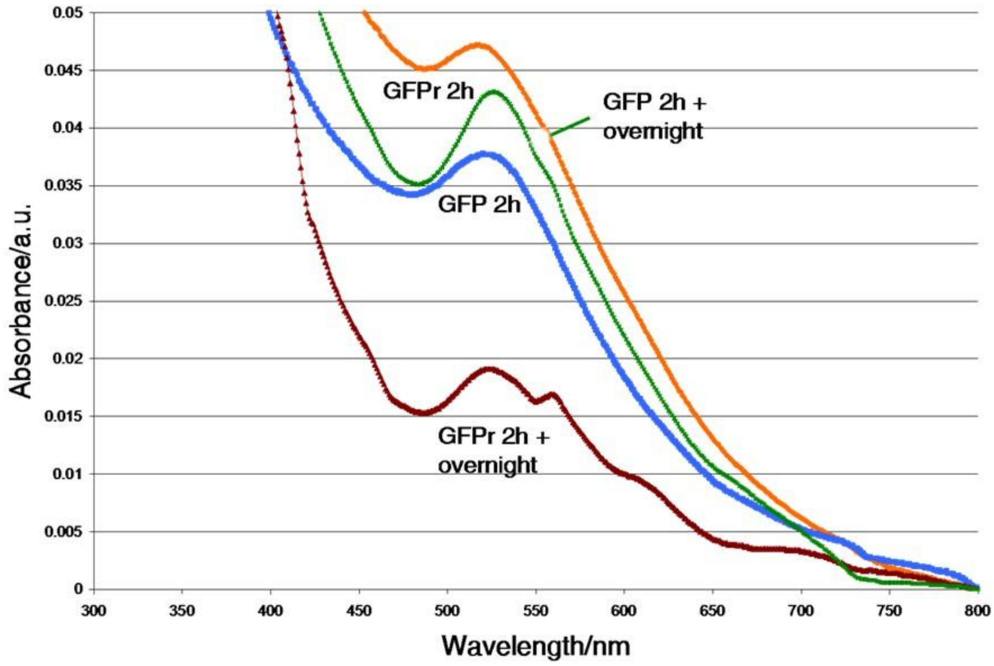

Figure SM-5. UV-Visible spectra of samples from Figure SM-3 (Panel A): lane 10 (GFP, 4 h incubation time), lane 11 (GFPr, 4 h), lane 12 (GFP, 7 h), and lane 14 (GFPr 7 h) incubated with gold phosphine at 70 ^{o}C. The emergence of spectral shoulders of GFPr 4 h and GFPr 7 h confirms the necessity of a specific initial DNA topological distribution to ensure narrow size-dispersity of the Au NP.

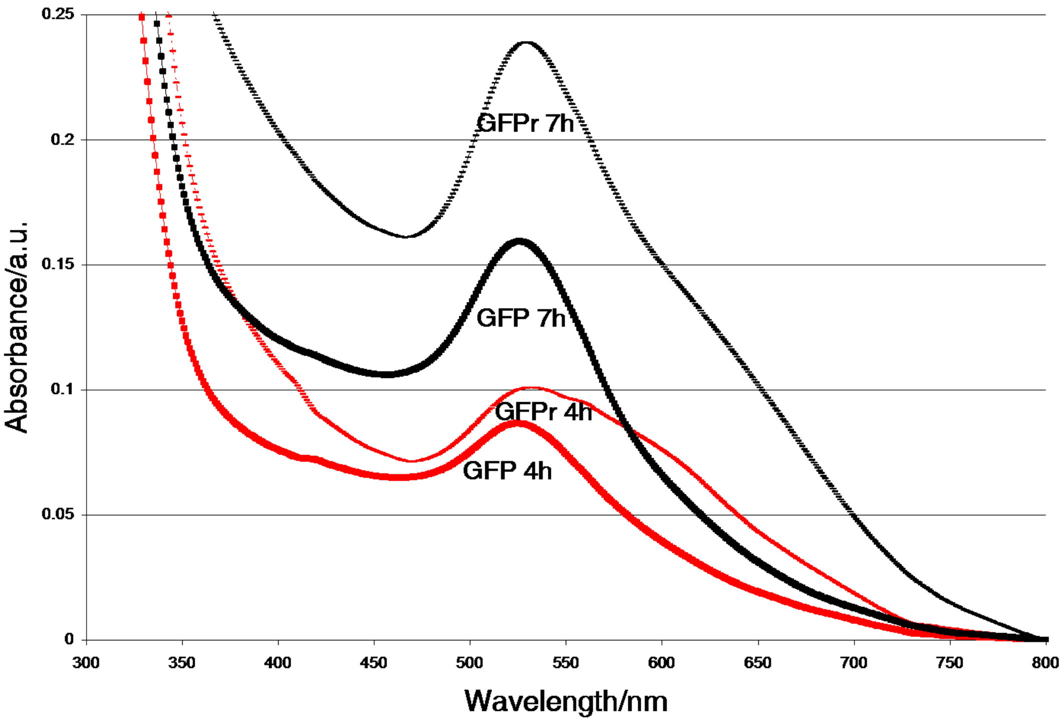

Figure SM-6. UV-visible spectra of samples incubated for 1 h and 7 h after DNA precipitation with isopropyl-alcohol. This result confirms that the NPs are bound on the DNA template after 1 hour of incubation (see also TEM micrographs in Figure 2) and that the DNA fragments, formed upon template degradation, act as anti-aggregating agents. The scattering due to the precipitated DNA results in significant background and the lack of signal due to the Au NP indicates they are in the precipitate.

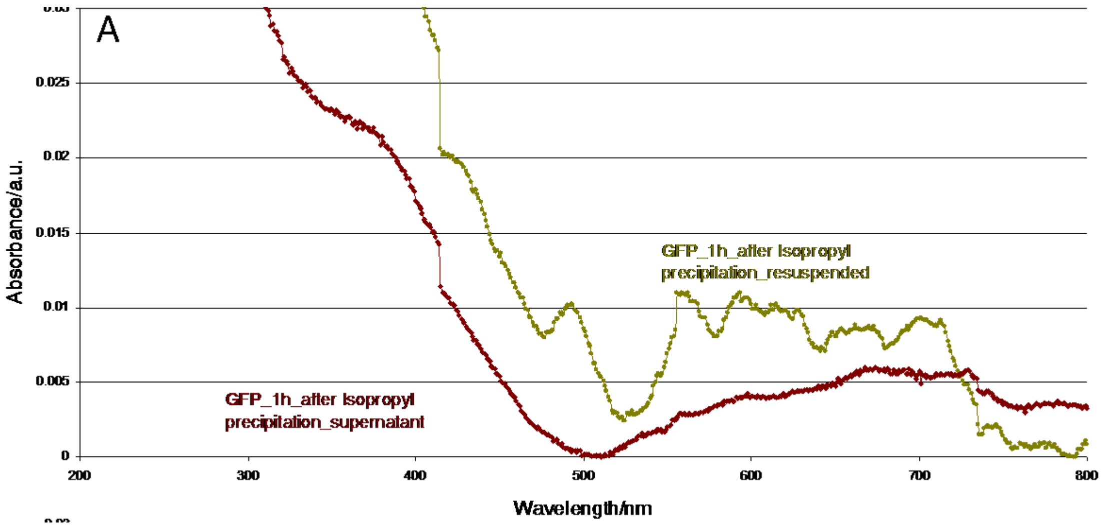

Figure SM-6. *Cont.*

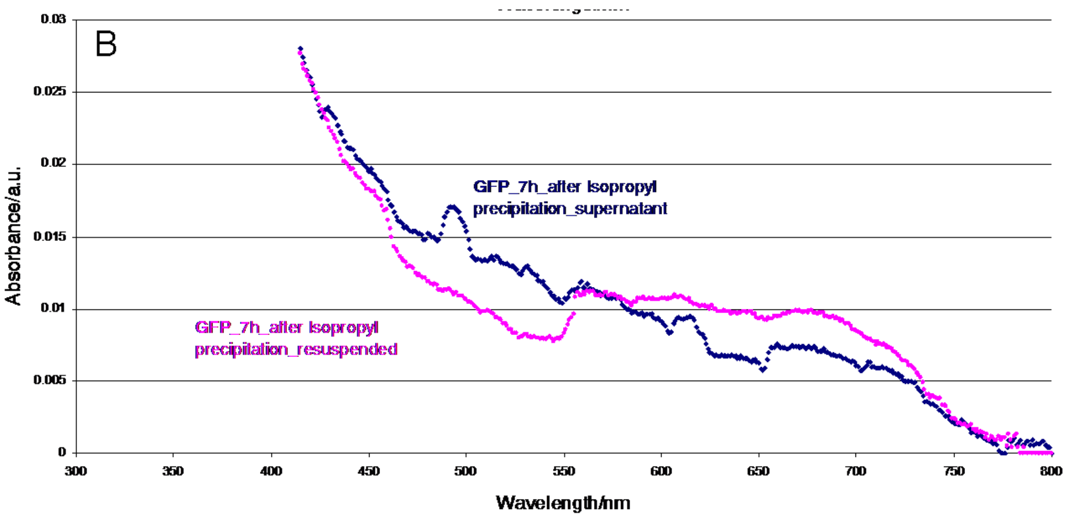

Figure SM-7. Panel A. Histogram illustrating Au NP size distribution after incubating GFP with the Au phosphine for 30 min. Panel B. TEM image showing that particles are still bound to DNA strands and the bimodal distribution suggests that the DNA major and minor grooves may act as enucleating sites and the overall function of the DNA is that of a bio-molecular reactor.

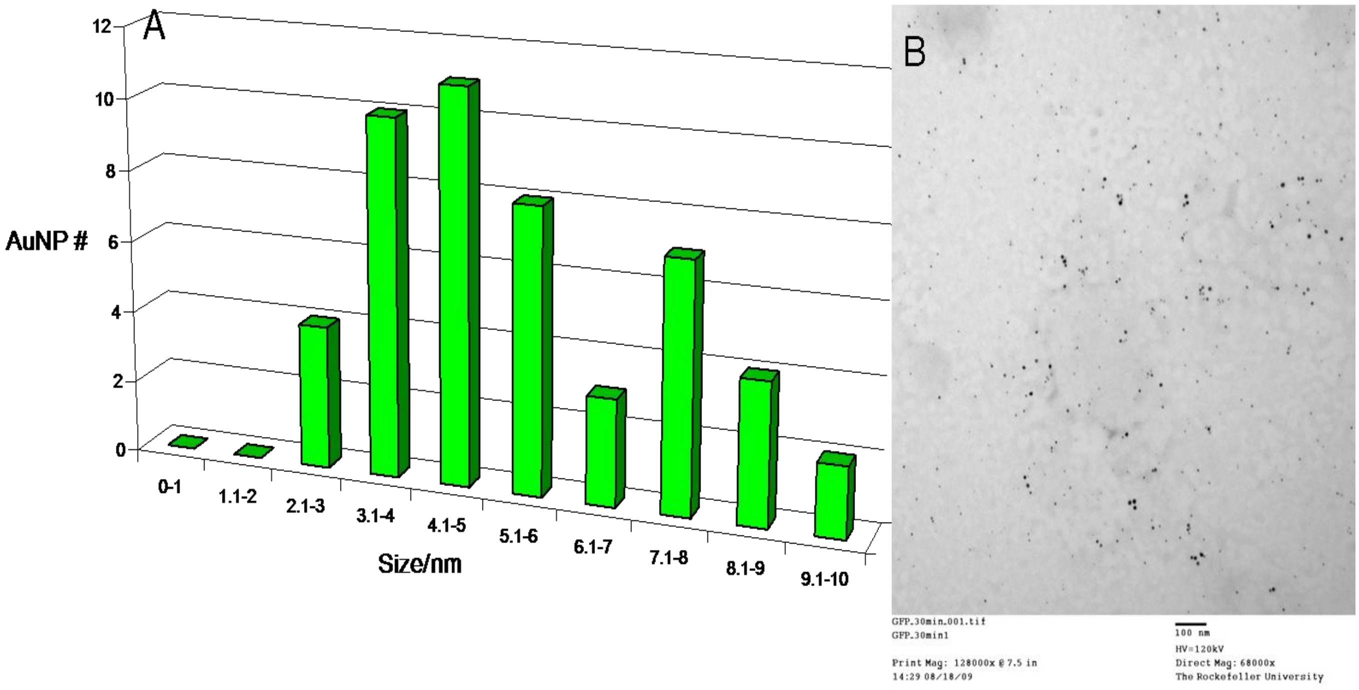

Figure SM-8. UV-Visible spectra of Au NP prepared from 50-fold diluted, and 5-fold more concentrated GFP suspensions, incubated with gold phosphine for 4 h at 70 ^{o}C. The broadening of the spectra as well as the lower absorbance of the 5-fold more concentrated sample confirms that narrow distribution is strongly dependent on the initial DNA concentration; the relative proportions of the gold ions, the Tris, and DNA are necessary for optimized size-control. This was found empirically.

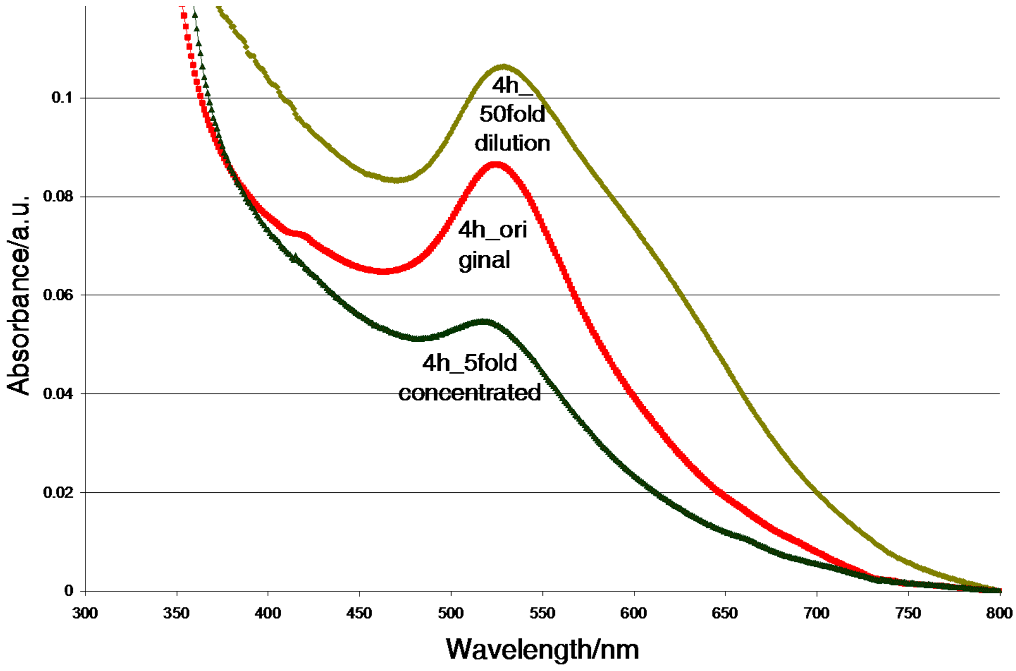

Figure SM-9. UV-visible spectrum of DNA suspension incubated with gold phosphine solution at 70 ^{o}C for 21 hour until the whole aqueous solution evaporated and condensed on top of the PCR Eppendorf tube. This spectrum shows that in order to fabricate NPs and maintain their stability, an aqueous medium is necessary.

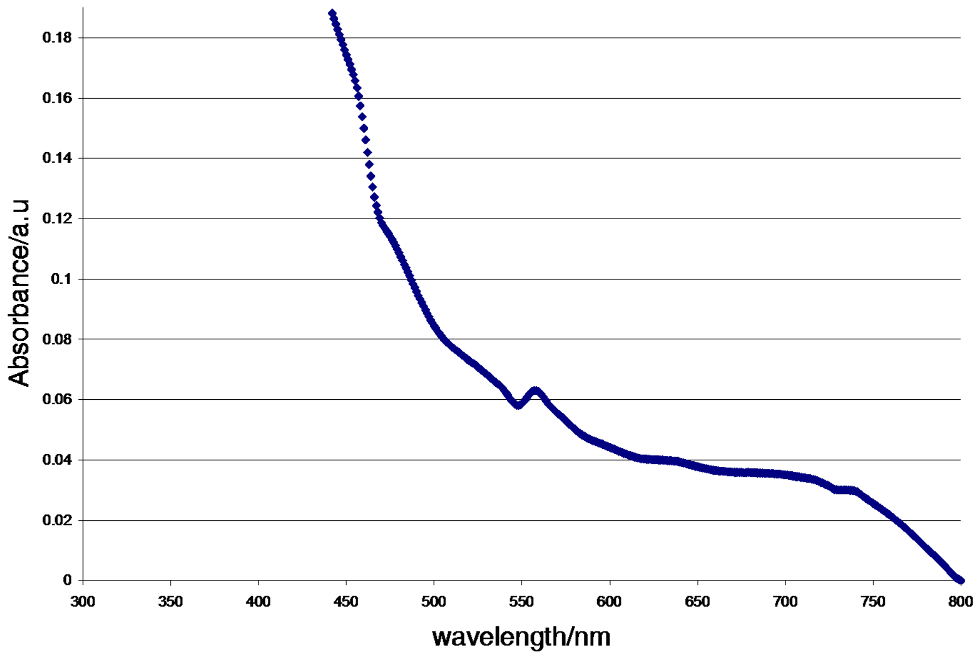

Supplement: Supplementary file 1 [file nanomaterials-01-00064-s001.pdf]
